# Supplementary material for: Peptidoglycan biosynthesis is driven by lipid transfer along enzyme-substrate affinity gradients
Source: Nat Commun. 2022 Apr 27;13:2278. doi: 10.1038/s41467-022-29836-x (PMC9046198; doi:10.1038/s41467-022-29836-x)
Supplement: Supplementary file 1 — Supplementary Information [file 41467_2022_29836_MOESM1_ESM.pdf]

SUPPLEMENTARY INFORMATION

Peptidoglycan biosynthesis is driven by lipid transfer along enzyme-substrate affinity gradients

Oluwole, A.O. et al.

TABLE OF CONTENTS

Supplementary Fig. 1 MS/MS spectra of MraY released from OG micelles. 2
Supplementary Fig. 2 Native mass spectra of MraY in different detergents. 3
Supplementary Fig. 3 Dimeric MraY preferentially binds to ligands. 4
Supplementary Fig. 4 MD simulations of MraY and C_{55}-P. 5
Supplementary Fig. 5 Poses of lipid I around MraY 6
Supplementary Fig. 6 Relative C55-P binding affinity of wild-type and Q260/R340 MraY measured in LDAO. 7
Supplementary Fig. 7 Identification of endogenous ligands bound to MraY. 8
Supplementary Fig. 8 Lipid II binding affinity of MurG. 9
Supplementary Fig. 9 Details of CG-MD simulations of MurG and lipid I and lipid II parametrisation. 10
Supplementary Fig. 10 MraY, MurG and MurJ bind their native lipid substrates more favourably than their products. 12
Supplementary Table 1: Masses of species observed in spectra shown in Fig. 1a. 13
Supplementary Table 2: Masses of species observed in the spectra shown in Figs. 2,3 and 5. 14
Supplementary Table 3: The k_{off} (μs^{-1}) for the C_{55}-P binding residues on the dimer interface of MraY. 15
Supplementary Table 4: Relative occupancies of C_{55}-P and lipid I calculated from the simulation of MraY dimer in a model lipid bilayer containing both C55-P and lipid I. 15
Supplementary Table 5: Oligonucleotide primers used to generate MraY mutants. 15
Supplementary references 15

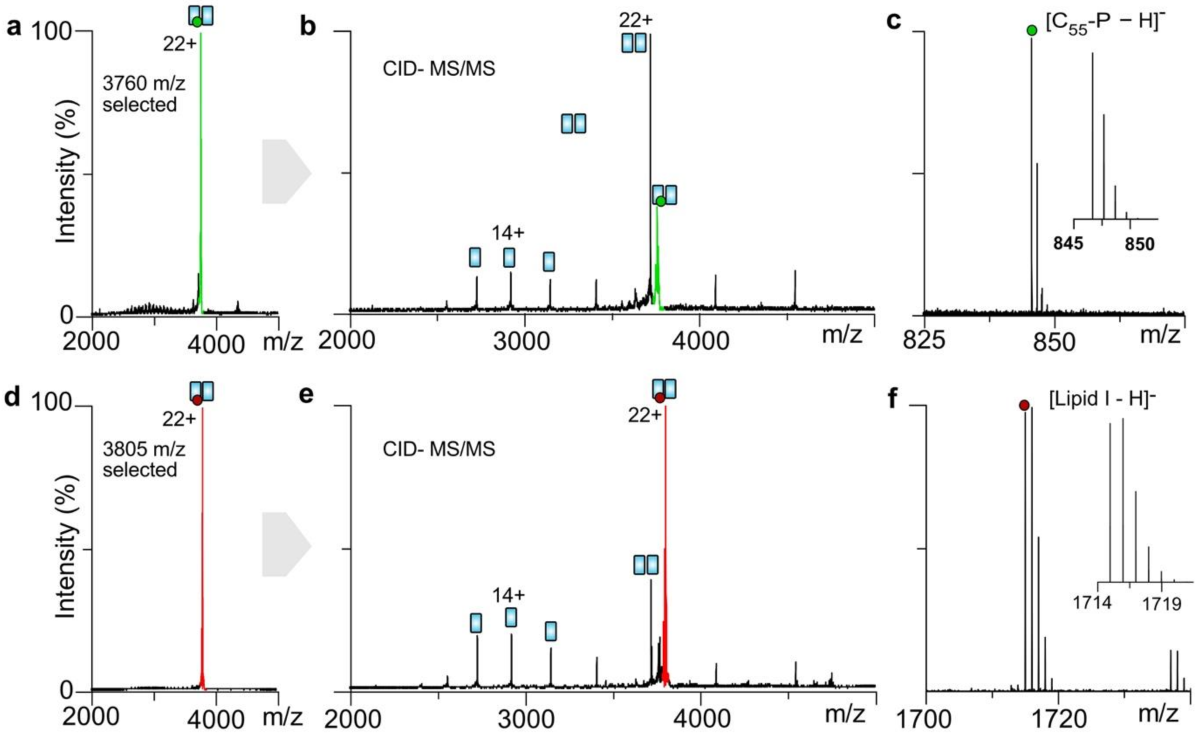

Supplementary Fig. 1 MS/MS spectra of MraY released from OG micelles. **a-c** Protein ions were released at the source region of the mass spectrometer with 300 V and the peak assigned to C_{55}-P bound MraY dimer was isolated (panel a) and subjected to collision-induced dissociation (CID) at 150 V (panel b). The ligand bound dimer dissociated into monomers and apo dimer that readily fall apart into monomers, the ejected ligand is detected in the negative mode by reversing the polarity and tuning the ion optics accordingly to enhance transmission and detection of low m/z ions (panel c). **d-f** The same sequence of experiments performed on the peak assigned to dimer MraY bound to lipid I. Theoretical isotopic distributions of C_{55}-P and lipid I shown as inserts in panels **c** and **f** are calculated using an online tool (https://prospector.ucsf.edu/prospector/cgibin/msform.cgi?form=msisotope). The observed isotopic pattern closely matches the theoretical isotopic distributions. For C_{55}-P [$(C_{55}H_{90}O_4P)^-$], the observed m/z = 845.6627 agrees with the calculated monoisotopic m/z = 845.6571 (mass deviation of 6.6 ppm). For the endogenous lipid I [$(C_{87}H_{142}N_7O_{23}P_2)^-$], the observed m/z = 1714.9749 Da while the calculated monoisotopic m/z = 1714.9627 (mass deviation of 7.1 ppm).

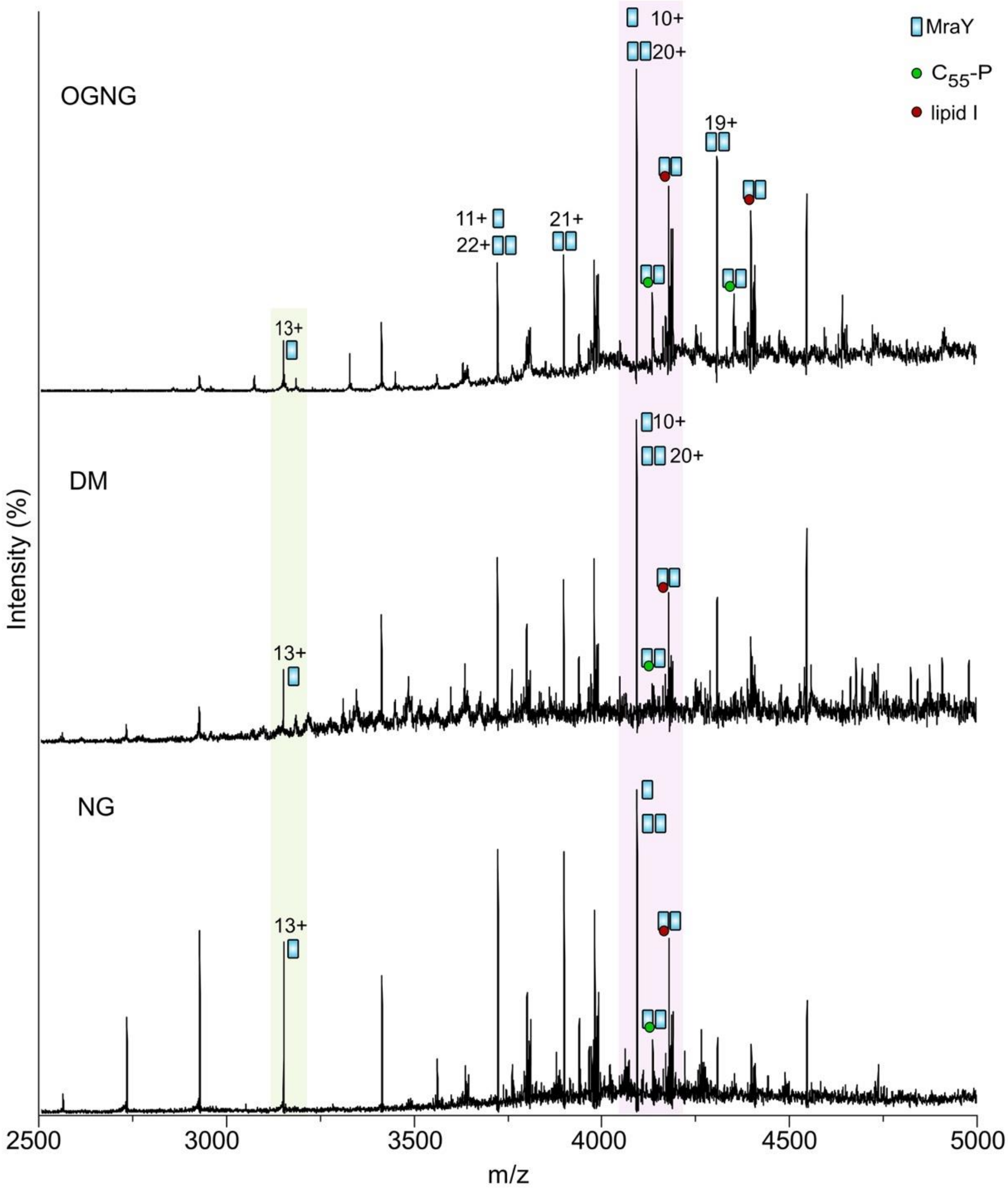

Supplementary Fig. 2 Native mass spectra of MraY in different detergents. a Spectra of MraY liberated from buffer containing 200mM ammonium acetate (pH8.0) and supplemented with 0.12% OGNG (2,2-dihexylpropane-1,3-bis-*β*-D-glucopyranoside), 0.4% NG (n-nonyl-*β*-D-glucopyranoside), or 0.17% DM (n-decyl-*β*-D-maltopyranoside). Peaks corresponding to monomers, apo dimers, and dimers (blue rectangle) bound to endogenous C_{55}-P (green circle) or lipid I (red circle) are labelled.

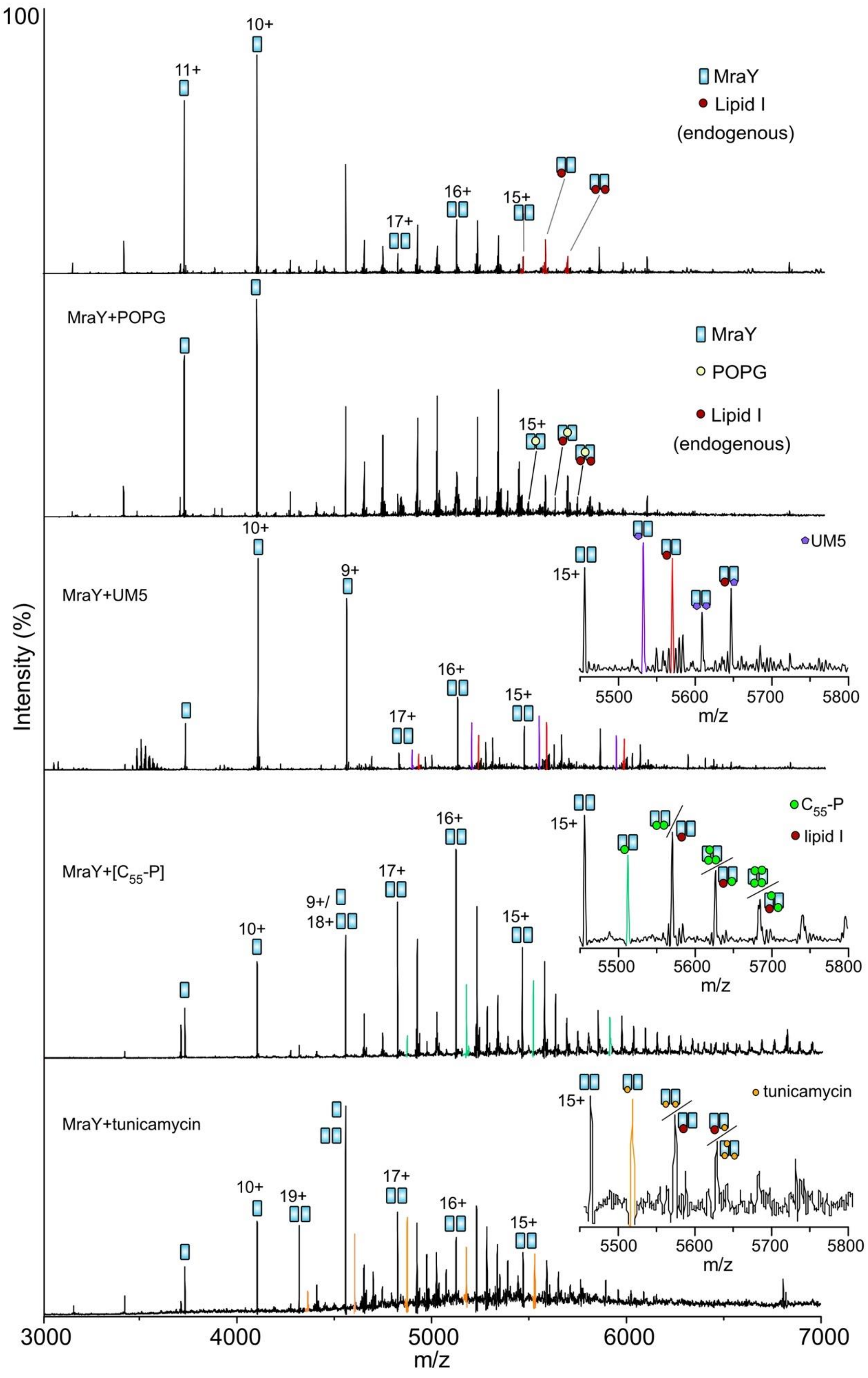

Supplementary Fig. 3 Dimeric MraY preferentially binds to ligands. Native MS of wild-type MraY incubated without ligands (top) and with a 10-fold molar excess of POPG, UDP-

MurNAc pentapeptide (L-Ala, D-Glu, L-Lys, D-Ala, D-Ala) (UM5), undecaprenyl phosphate (C_{55}-P), or tunicamycin. Protein-ligand solutions were equilibrated overnight at 4 ^{o}C in a buffer containing 0.02% DDM, 20 mM Tris (pH8.0), 200 mM NaCl, 10% glycerol and subsequently exchanged into 200 mM ammonium acetate and 0.5% C8E4 prior to MS to remove excess ligands. Inserts, dimer (15+) charge states. Selected peaks are corresponding exclusively to dimer-bound UM5 (purple), C_{55}-P (green), tunicamycin (yellow) or endogenous lipid I (red), are highlighted. Spectra were acquired using the same instrument settings, 200 V in the HCD.

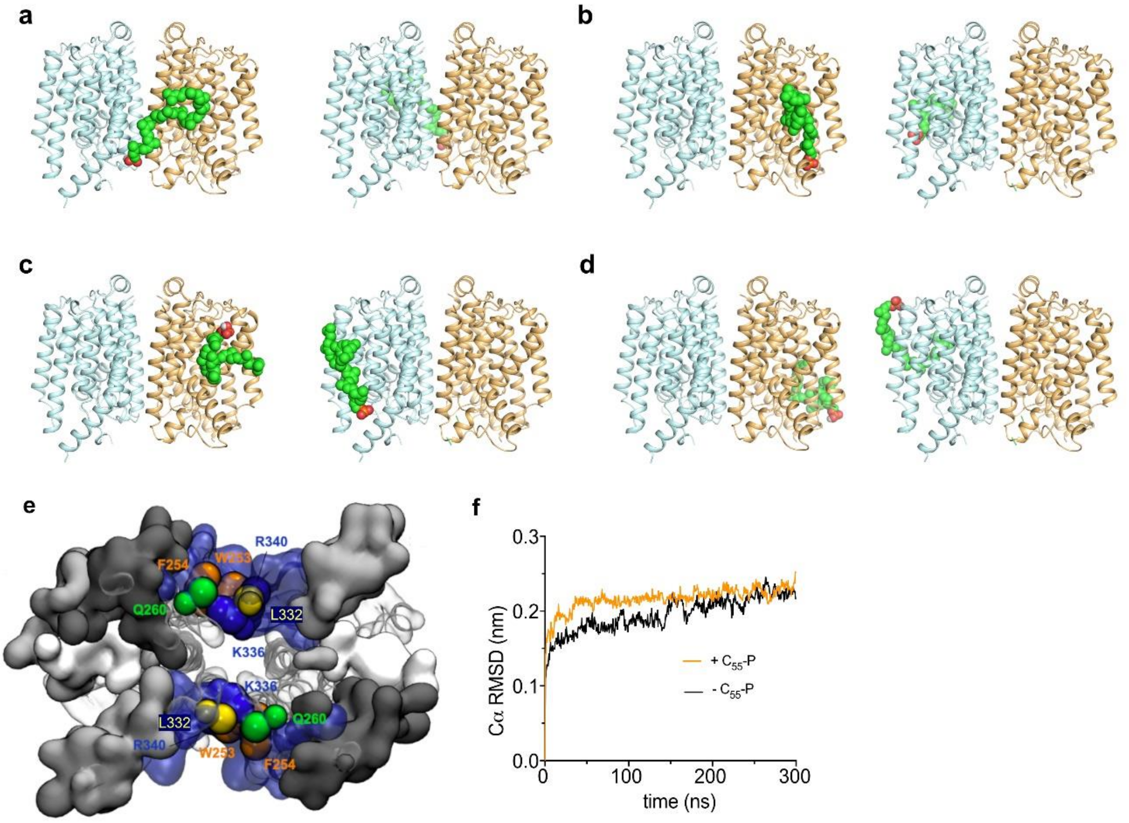

Supplementary Fig. 4 MD simulations of MraY and C_{55}-P. a-d Representative pose of C_{55}-P bound to MraY from atomistic molecular dynamic simulations. Panels **a** shows C_{55}-P binding to the dimer interface, **b** to the active site, while **c** and **d** show a selected lower affinity poses around the MraY dimer. The protein backbone chain is shown as a cartoon, with one protomer coloured gold and the other cyan. A C_{55}-P molecule is shown as coloured spheres (hydrophobic tail, green). **e** The C_{55}-P binding sites around the MraY dimer interface, as identified using network analysis on CG MD data with the PyLipID program[1]. Only cytoplasmic C_{55}-P binding sites with the lipid substrate present for >50% of the MD frames are shown. The equivalent sites on each side of the MraY dimer are shown in the same colour. The sites with the highest affinity are shown in blue with the principal contributing residues shown as coloured spheres. Calculated k_{off} of 1.27 ± 0.35 μs^{-1} and 0.94 ± 0.39 μs^{-1} are obtained for the blue sites with values and errors based on 10 rounds of bootstrapping. **f** RMSDs of MraY $C\alpha$ atoms over 3 x 300 ns of atomistic simulation either with (+) or without (-) C_{55}-P present. Data are the mean of 3 repeats.

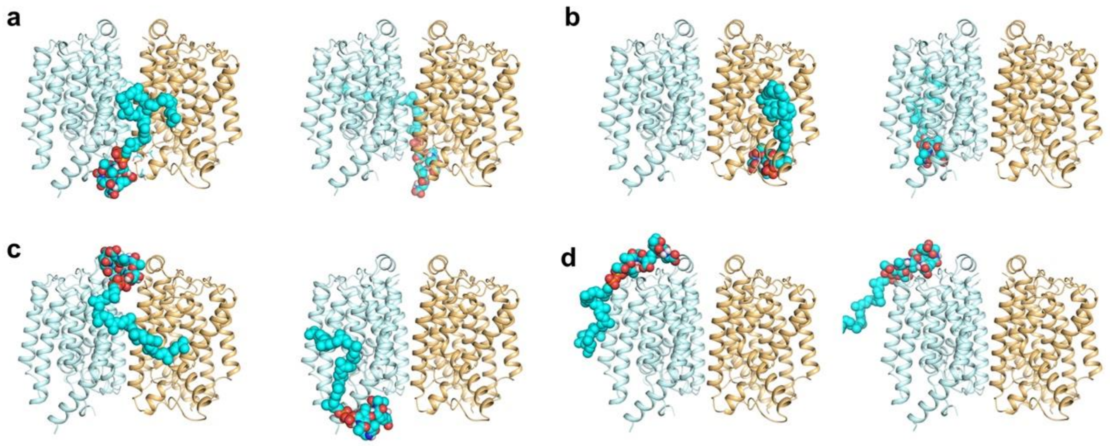

Supplementary Fig. 5 Poses of lipid I around MraY. a-d A number of lipid I binding poses on the MraY dimer generated for lipid I from the simulation data using PyLipID[1], and converted to atomic detail for visualisation using CG2AT [2]. Panels: **a** shows binding to the dimer interface, **b** to the active site, while. **c** and **d** show a select few of alternative lower affinity binding poses of lipid I around the MraY dimer.

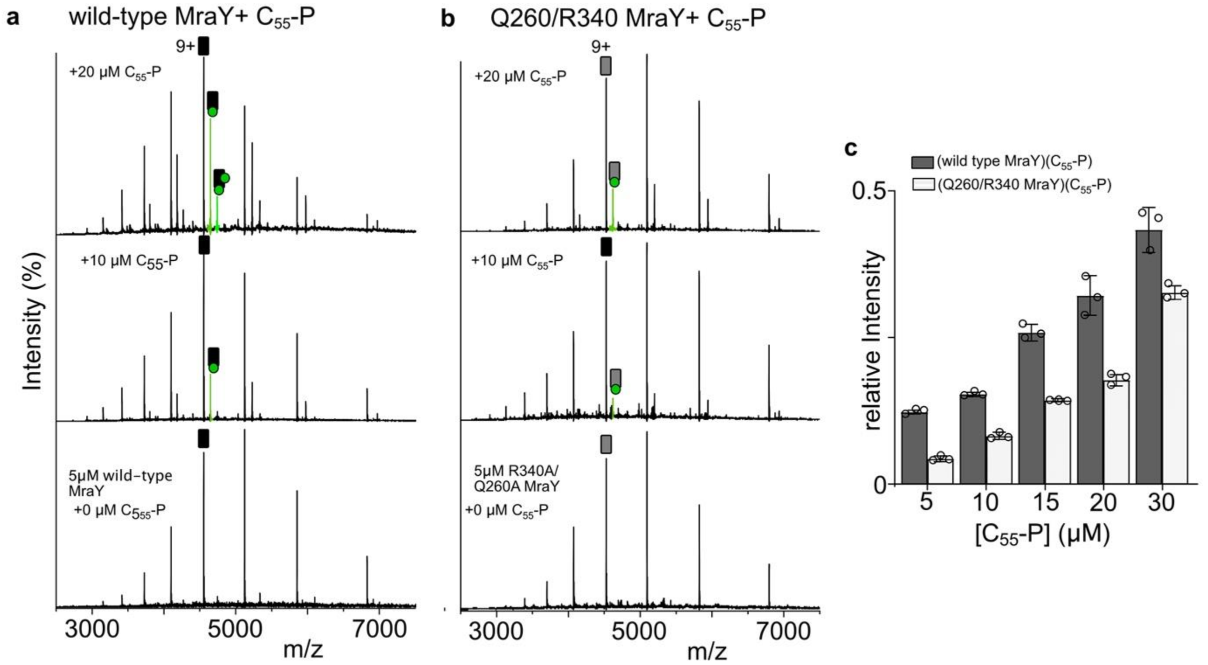

Supplementary Fig. 6 Relative C55-P binding affinity of wild-type and Q260/R340 MraY measured in LDAO. Representative Native MS of **a** 5 µM wild type MraY and **b** 5 µM Q260A/R340A MraY titrated with C_{55}-P**.** MraY was buffer exchanged into 200 mM ammonium acetate, 0.05% LDAO and aliquots were titrated with indicated concentrations of C_{55}-P in the same 'MS buffer'. Samples were incubated for 20-30 min prior to the acquisition of mass spectra, activation voltage of 100 V. Protein-bound C_{55}-P is denoted by green circle. At protein: ligand molar ratios (from 0 to 4), wild-type MraY formed 1:1 and 1:2 protein/ligand complexes, while only the 1:1 complex was observed for the mutant. Spectra shown are representative of 3 independent measurements. **c** Relative affinity of wild-type and Q260A/R340A MraY bound to C_{55}-P. Data are presented as the mean relative intensities (± SD) of ligand-bound proteins, with $n = 3$; while each circle represents individual measurement value. Source data are provided as a Source data file.

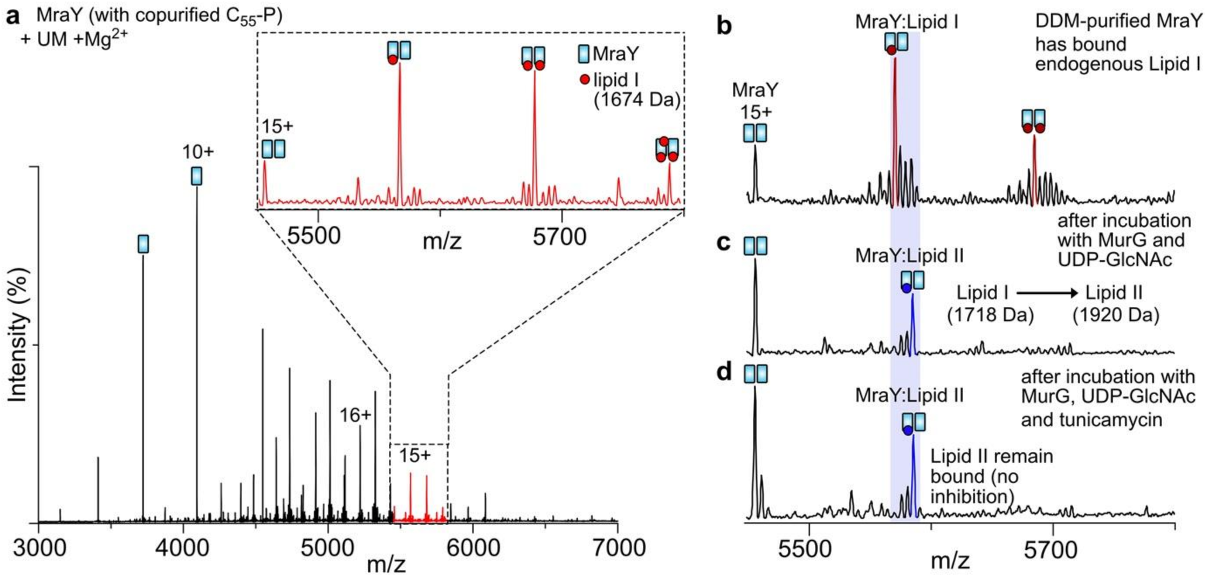

Supplementary Fig. 7 Identification of endogenous ligands bound to MraY. a Mass spectrum of MraY purified in DDM and incubated overnight with a 10-fold molar excess of UDP-MurNAc-pentapeptide (UM5) and 2 mM Mg^{2+}. Endogenous C_{55}-P that copurified with MraY reacts with UM5, the resulting lipid I products can be seen as three series of 1674-Da adduct bound to dimeric MraY. Insert, an expanded view of the dimer charge state (15+). **b** Mass spectrum of MraY (dimer 15+ charge state) after overnight incubation with Mg^{2+} in SEC buffer (containing 0.02% DDM). The peaks shown correspond to dimer MraY (81836.38±0.85 Da) and dimer bound to one or two endogenous lipid I molecules (83554.25±0.25 Da, 85270.51±0.55 Da). Thus, a molecule of bound endogenous lipid I has an average mass of 1717.47 Da. **c** Spectrum of MraY after incubation with Mg^{2+}, MurG, and UDP-GlcNAc. Peaks in this spectrum correspond to apo dimer (81836.55±0.51 Da) and lipid II bound dimer (83757.92±0.20 Da). The mass difference between these species correspond to 1921.54 Da, and is equivalent to an additional mass of 203.7 Da over that of endogenous lipid I. The mass 203.7 Da corresponds closely to a GlcNAc residue added by MurG to lipid I. **d** A reaction mixture of composition as in **b** but in the presence of 250 μM tunicamycin. Tunicamycin does not inhibit the conversion of lipid I into lipid II. Of note, MurG was not observed in these spectra due to its instability in the C8E4-containing buffer used for the measurements (the reaction itself was performed in a buffer containing 0.02% DDM, 20 mM Tris (pH8.0), 200 mM NaCl, 10% glycerol and 2 mM $MgCl_2$.

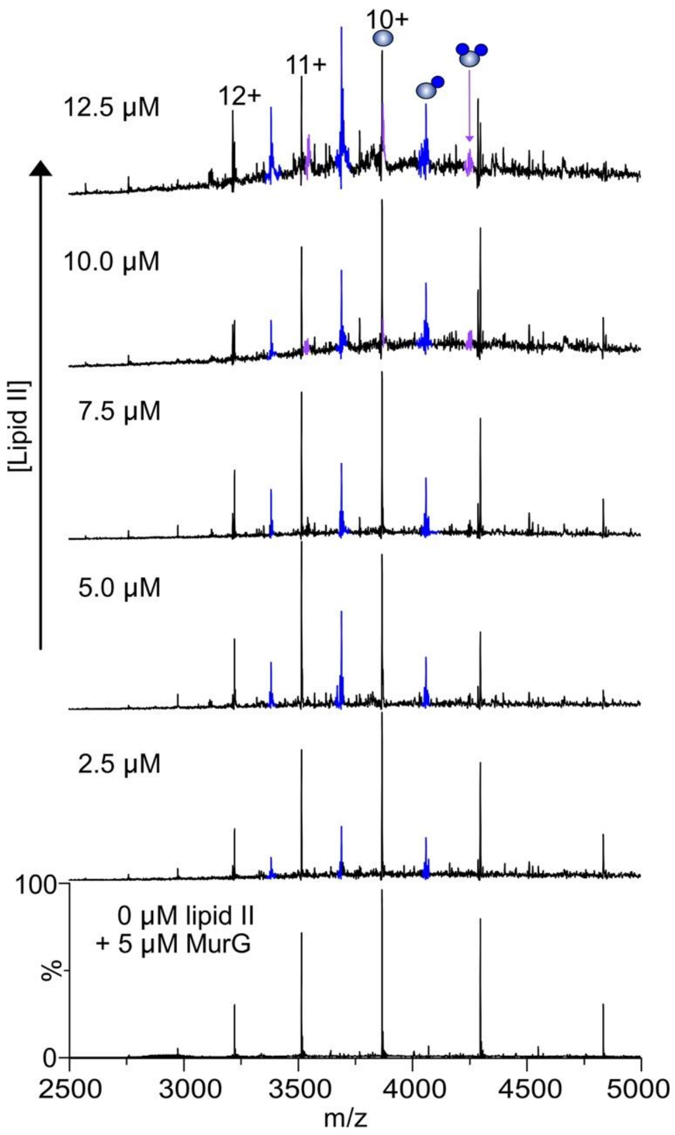

Supplementary Fig. 8 Lipid II binding affinity of MurG. Shown are the representative mass spectra for 5 µM MurG titrated with increasing concentrations of lipid II. Proteins were released from a buffer containing 200 mM ammonium acetate, 0.05% LDAO, pH 8.0 with an activation voltage of 150 V. The same condition was used for MurG-lipid I binding data (Figure 4a).

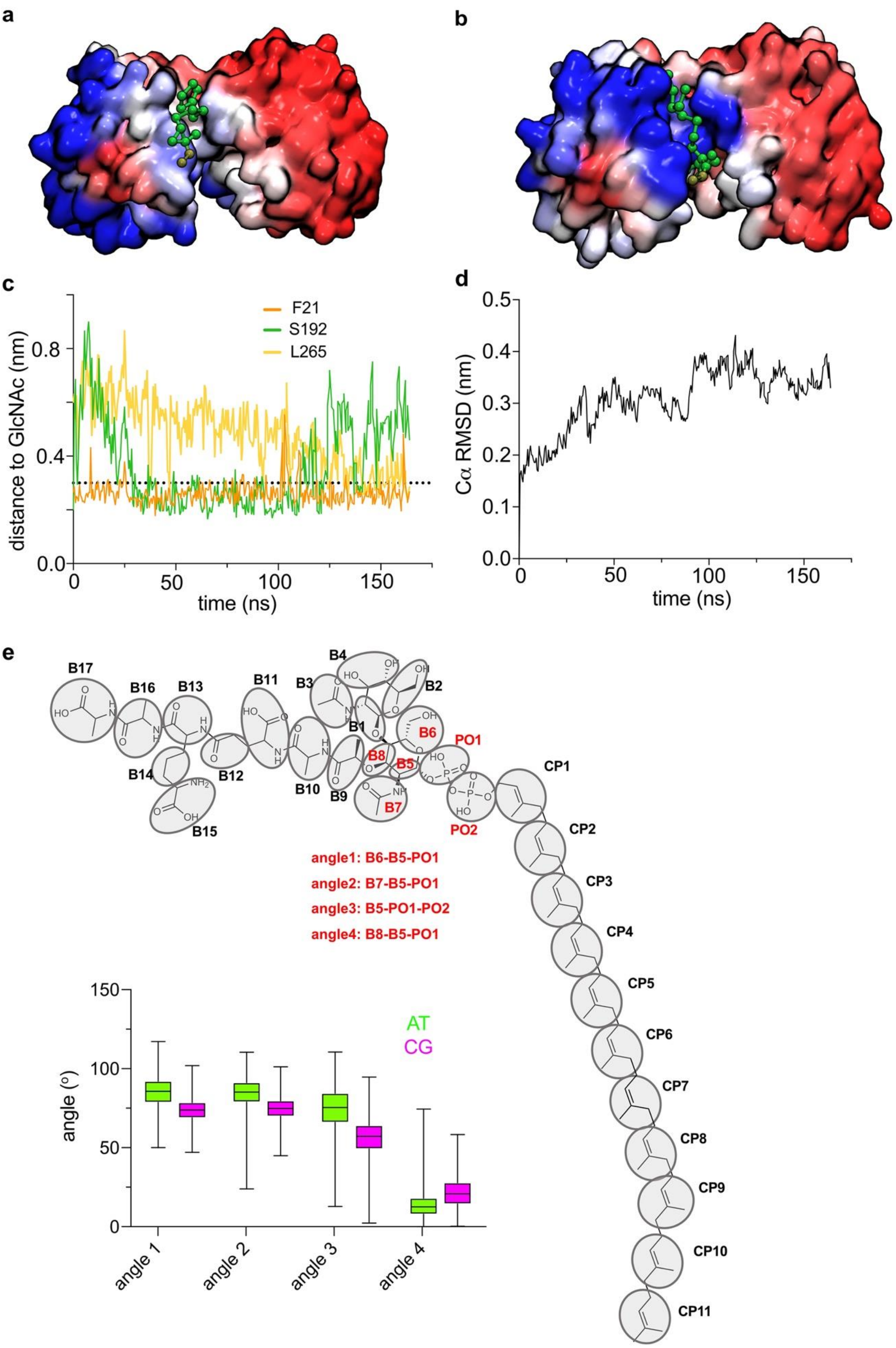

Supplementary Fig. 9 Details of CG-MD simulations of MurG and lipid I and lipid II parametrisation. a,b Headgroup binding pose of **a** lipid I and **b** lipid II bound to MurG, taken

from CG, as produced by PyLipID. Colour scale is occupancy (i.e.% of simulation time bound), from 0-50% (red>blue). **c** Distance between MurG residues Phe-21, Ser-192 and Leu-265 (highlighted in Figure 4d) and the GlcNAc of lipid II in the atomistic simulation. The approximate distance of these residues to GlcNAc in the atomic structure of MurG (PDB code: 1NLM) is shown as a dotted line**. d** RMSD of Cα from 160 ns of atomistic MD. **e** Mapping details for lipid II in CG. Shown is the chemical structure of lipid II, with circles marking the beads used for the Martini 2 parameters. Each bead is labelled with its name in parameters, available at https://doi.org/10.6084/m9.figshare.19403852v2. The bonded terms in the connecting region, highlighted in red, were parameterized according to atomistic data. Note that the parameters for lipid I are identical, but without beads B1, B2, B3 and B4. Inset, a box plot showing comparisons of the connecting angles from a CG (green) and atomistic MD (magenta) simulation, n=30,000 snapshots for the atomistic and n=15,000 snapshots for the CG. The box plot represents the median (middle line) and the 25th and 75th percentiles while the error bars show the maximum and minimum values. Source data are provided as a Source data file.

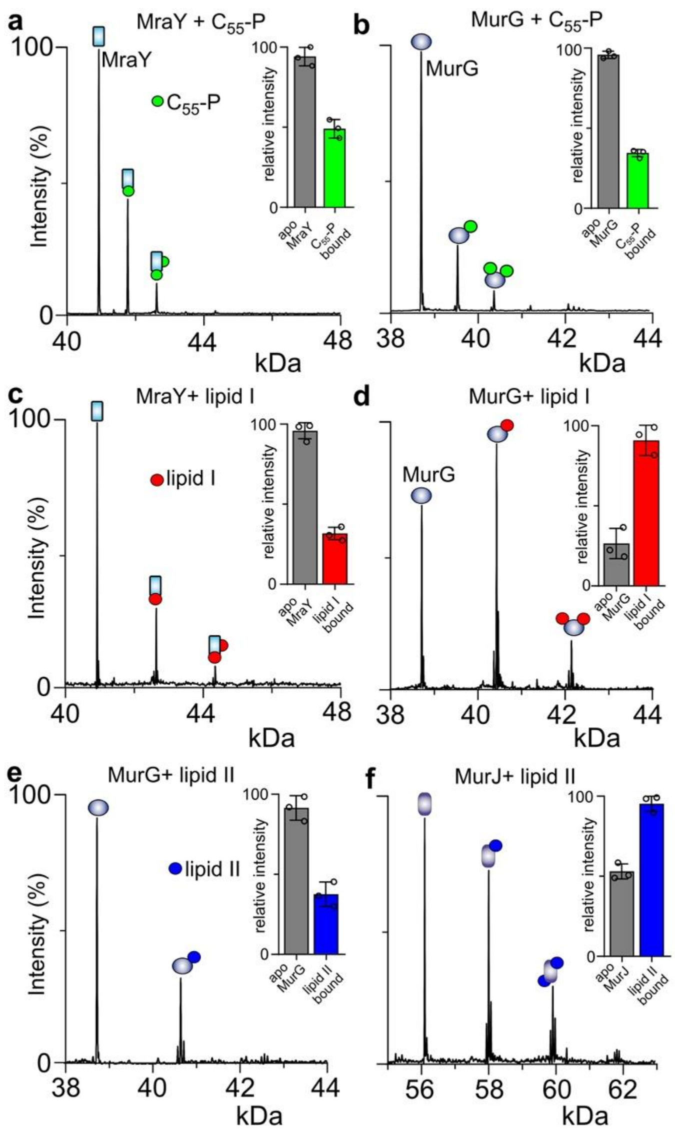

Supplementary Fig. 10 MraY, MurG and MurJ bind their native lipid substrates more favourably than their products. Shown are the deconvoluted mass spectra for solutions containing **a** 5 µM MraY and 20 µM C_{55}-P, **b** 5 µM MurG and 20 µM C_{55}-P, **c** 5 µM MraY and 5 µM lipid I, **d** 5 µM MurG and 5 µM lipid I, **e** 5 µM MurG and 5 µM lipid II, and **f** 5 µM MurJ and 5 µM lipid II. The carrier lipid substrate C_{55}-P binds more favourably to MraY than to MurG. Lipid I binds more favourably to MurG than to the MraY. Lipid II binds more favourably to MurJ than to MurG. Inserts, the mean (± SD) relative intensities of ligand-free and ligand-bound proteins for *n*=3 independent measurements. Each circle represents individual measurement value. Source data are provided as a Source data file.

Supplementary Table 1: Masses of species observed in spectra shown in Fig. 1a. (C55-P, undecaprenyl phosphate; C55-PP, undecaprenyl diphosphate; "– 1×Ala" and "– 2×Ala" respectively indicate lipid I and lipid II variants without the terminal 1 and 2 alanine residues in the peptide stem. The average theoretical masses of MraY, C55-P, C55-PP, lipid I, and lipid II are 40919 Da, 847 Da, 927 Da, 1717 Da, and 1920 Da, respectively.

Observed Mass (Da)	Assignments
40918.03±0.47	$(MraY)_1$
81836.18±0.96	$(MraY)_2$
82683.52±1.07	$(MraY)_2(C_{55}\text{-P})$
82762.42±1.74	$(MraY)_2(C_{55}\text{-PP})$
83239.08±0.87	$(MraY)_2$(cardiolipin)
83371.92±0.54	not assigned
83412.89±1.56	$(MraY)_2$(lipid I – 2×Ala)
83483.23±0.64	$(MraY)_2$(lipid I – 1×Ala)
83553.36±0.28	$(MraY)_2$(lipid I)
83617.00±0.65	$(MraY)_2$(lipid II – 2×Ala)
83685.30±1.32	$(MraY)_2$(lipid II – 1×Ala)
83756.12±2.02	$(MraY)_2$(lipid II)
84401.85±0.86	$(MraY)_2$(lipid I)$(C_{55}\text{-P})$
84997.11±2.41	Residual MBP-MraY
85268.96±0.55	$(MraY)_2$(lipid I)$_2$

Supplementary Table 2: Masses of species observed in the spectra shown in Figs. 2,3 and 5.

Mass (Da)		Assignments
Experimental	Theoretical	
Fig. 2a and Supplementary Fig. 5 (MraY monomeric species)		
40918.03±0.47	40919	Wild-type MraY
40860.25±0.16	40862	Q260A MraY
40860.37±0.19	40862	K336A MraY
40832.64±0.39	40834	R340A MraY
40775.07±0.17	40777	Q260A/R340A MraY
40641.92±0.48	40642	W253A/F254A/R340A MraY
Fig. 3a (lower panel)		
40918.03±0.15	40919	MraY
81836.25±0.72	81838	$(MraY)_2$
83509.64±0.36	83510	$(MraY)_2$(lipid I)
85182.42±0.22	85184	$(MraY)_2$(lipid $I)_2$
86855.76±0.49	86858	$(MraY)_2$(lipid $I)_3$
Fig. 5a		
38693.52±0.35	38693	MurG
39618.62±0.29	39620	(MurG)(C_{55}-PP)
40365.73±0.54	40367	(MurG)(lipid I)
40917.39±0.52	40918	MraY
42039.43±0.18	42041	(MurG)(lipid $I)_2$
42590.63±0.30	42592	(MraY)(lipid I)
Fig. 5b		
38693.12±0.15	38693	MurG
40365.45±1.43	40367	(MurG)(lipid I)
40568.92±0.86	40570	(MurG)(lipid II)
40917.14±0.87	40918	MraY
42040.33±0.54	42041	(MurG)(lipid $I)_2$
42242.57±0.43	42244	(MurG)(lipid I)(lipid II)
42446.09±0.18	42447	(MurG)(lipid $II)_2$
42793.10±0.80	42795	(MraY)(lipid II)
Fig. 5c		
38694.00±0.12	38693	MurG
40917.69±0.47	40919	MraY
56032.26±0.26	56033	MurJ
57908.33±0.44	57909	(MurJ)(lipid II)

Supplementary Table 3: The k_{off} (μs^{-1}) for the C_{55}-P binding residues on the dimer interface of MraY.

Residues	Arg-340	Leu-332	Trp-253	Lys-336	Phe-254	Gln-260
protomer 1	2.653	3.185	2.494	5.025	5.814	0.118
protomer 2	2.128	2.179	3.125	5.464	6.623	9.709

Supplementary Table 4: Relative occupancies of C_{55}-P and lipid I calculated from the simulation of MraY dimer in a model lipid bilayer containing both C55-P and lipid I.

Ligand	Occupancies (%)		
	Interfacial sites	Catalytic sites	
C_{55}-P	71.1; 67.3	37.6; 25.3	
Lipid I	37.6; 25.3	75.1; 81.3	

Supplementary Table 5: Oligonucleotide primers used to generate MraY mutants.

Name	Sequence
Q260A_Rev	AACATTGCAGCCGGGAAGGAGTTGAACC
Q260A_Fwd	CCCGGCTGCAATGTTTATGGGGGATGTGGGTAGC
K336A_Rev	ACAATGGCCGGTTCAGGCAGACCATTCA
K336A_Fwd	TGAACCGGCCATTGTTGTCCGTATGTGGATTATT
R340A_Rev	CACATAGCGACAACAATTTTCGGTTCAGGC
R340A_Fwd	TGTTGTCGCTATGTGGATTATTAGCATCCTGC
W253/F254_Rev	AGTTGGCCGCCAGAAACCCCAGACCAGCAC
W253/F254_Fwd	TTCTGGCGGCCAACTCCTTCCCGGCTCAAATG

Supplementary references

1. Song, W. et al. PyLipID: A Python Package for Analysis of Protein-Lipid Interactions from Molecular Dynamics Simulations. *J. Chem. Theory Comput.* **18**, 1188–1201 (2022).
2. Vickery, O.N. & Stansfeld, P.J. CG2AT2: an Enhanced Fragment-Based Approach for Serial Multi-scale Molecular Dynamics Simulations. *J. Chem. Theory Comput.* **17**, 6472-6482 (2021).
